# Validity and reliability of the Functioning Assessment Short Test (FAST) in bipolar disorder

**DOI:** 10.1186/1745-0179-3-5

**Published:** 2007-06-07

**Authors:** Adriane R Rosa, Jose Sánchez-Moreno, Anabel Martínez-Aran, Manel Salamero, Carla Torrent, Maria Reinares, Mercè Comes, Francesc Colom, Willemijn Van Riel, Jose Luis Ayuso-Mateos, Flávio Kapczinski, Eduard Vieta

**Affiliations:** 1 Bipolar Disorders Program & Molecular Psychiatry Laboratory, Hospital Clinic of Porto Alegre, Ramiro Barcelos, 2350, 90035-003, Porto Alegre, RS, Brazil; 2 Bipolar Disorders Program, Clinical Institute of Neuroscience, Hospital Clinic of Barcelona, Villarroel 170, Barcelona 08036, Barcelona, Spain; 3 Department of Psychiatry. Universidad Autonoma de Madrid. Hospital Universitario de la Princesa, Madrid, Spain

## Abstract

**Background:**

Numerous studies have documented high rates of functional impairment among bipolar disorder (BD) patients, even during phases of remission. However, the majority of the available instruments used to assess functioning have focused on global measures of functional recovery rather than specific domains of psychosocial functioning. In this context, the Functioning Assessment Short Test (FAST) is a brief instrument designed to assess the main functioning problems experienced by psychiatric patients, particularly bipolar patients. It comprises 24 items that assess impairment or disability in six specific areas of functioning: autonomy, occupational functioning, cognitive functioning, financial issues, interpersonal relationships and leisure time.

**Methods:**

101 patients with DSM-IV TR bipolar disorder and 61 healthy controls were assessed in the Bipolar Disorder Program, Hospital Clinic of Barcelona. The psychometric properties of FAST (feasibility, internal consistency, concurrent validity, discriminant validity (euthymic vs acute patients), factorial analyses, and test-retest reliability) were analysed.

**Results:**

The internal consistency obtained was very high with a Cronbach's alpha of 0.909. A highly significant negative correlation with GAF was obtained (r = -0.903; p < 0.001) pointing to a reasonable degree of concurrent validity. Test-retest reliability analysis showed a strong correlation between the two measures carried out one week apart (ICC = 0.98; p < 0.001). The total FAST scores were lower in euthymic (18.55 ± 13.19; F = 35.43; p < 0.001) patients, as compared with manic (40.44 ± 9.15) and depressive patients (43.21 ± 13.34).

**Conclusion:**

The FAST showed strong psychometrics properties and was able to detect differences between euthymic and acute BD patients. In addition, it is a short (6 minutes) simple interview-administered instrument, which is easy to apply and requires only a short period of time for its application.

## Background

*Kraepelin *in 1921 [1] noted that manic or depressive episodes were periodic in nature, and typically were followed by a return to what was then considered normal functioning. In contrast to early studies, recent studies do not describe such a favourable outcome in patients with bipolar disorder [2-5]. T*ohen et al*. (2000) [6] showed that although 97.5% of bipolar patients achieved syndromal recovery 24 months after admission, only 37.6% achieved functional recovery. *Strakowski et al*. (2000) [7], in a 8-month follow-up study, reported that nearly all of the remitted patients exhibited persistent impairment in at least one area of functioning and less than half achieved a good functional outcome in three of the four areas of functioning studied.

The concept of functioning is complex and involves many different domains including the capacity to work, capacity to live independently, capacity for recreation, capacity for romantic life, and capacity to study [4,6,8]. Researchers traditionally measure one or two elements of functioning and typically fail to take into account all the other elements necessary for optimal functioning. The measures used to assess functional impairment in BD varied greatly across studies, only a few instruments were used by more than two researchers [3,9,10]. Among multidimensional scales assessing functioning, the Global Assessment of Functioning scale (GAF) is the most commonly used, but the original GAF instructions call for rating symptoms as well as functioning [11-13]. Beyond these scales, the Social Adjustment Scale (SAS), the Life Functioning Questionnaire (LFQ), the Short Form-36 (SF-36) and the WHO-DAS are also used, but none were specific instruments developed to assess specific areas of functional impairment in bipolar disorder and required a longer time for its administration.

Future studies should be sensitive to the need for measures to evaluate the impact of illness factors on each domain of functioning [3] and the development of instruments that capture the specific issues related to severe mental illness and particularly BD are required [14]. The FAST (Functioning Assessment Short Test) was developed for the clinical evaluation of functional impairment presented by patients suffering from mental disorders including bipolar disorder. It is a simple instrument, easy to apply and that requires a very short time to be administered. The 24 items of the scale are divided among 6 specific areas of functioning: autonomy, occupational functioning, cognitive functioning, financial issues, interpersonal relationships and leisure time. Taking into consideration the opinion of experts the performance of previous scales and the literature, the aforementioned items were identified as the main problems experienced by the mentally ill, including bipolar patients [7,15-17].

The purpose of the present study was to validate the Spanish version of the FAST for its use as an instrument to assess functional impairment in subjects with bipolar disorder.

## Methods

### 1. Subjects

The study was conducted in the Bipolar Disorder Program, Hospital Clinic of Barcelona, Spain. One hundred and one bipolar patients were selected according to the Structured Clinical Interview for the DSM-IV TR criteria [11].

The study was approved by the Hospital Clinic of Barcelona Ethics Committee and was carried out in compliance with the Helsinki Declaration of 1975 (the Evaluation, Support and Prevention Unit).

### 2. Variables

After informing the patients and obtaining their consent, the investigator recorded their socio-demographic and clinical variables and administered the Spanish version of the Young Mania Rating Scale (YMRS) [18], Spanish version of the 17-item Hamilton Depression Rating Scale (HDRS-17) [19] and the Global Assessment Functioning (GAF) [20] to confirm the stability of the patient's condition and overall functioning. They also recorded all the medication prescribed to the patients for this visit. Finally, the investigator administered the FAST. Interviewers administering the FAST and the GAF were blinded to each other.

Sixty one control subjects were screened using the SCID (DSM-IV TR) to exclude current or lifetime psychiatric disorders. Controls had no first-degree relatives with bipolar disorder or other psychiatric disorders. The healthy comparison group was recruited from the general population within the catchment area of the Hospital Clinic, Barcelona, and gave written informed consent to participate in this study.

### 3. FAST

#### 3.1. Development

The FAST was developed by the Bipolar Disorder Program, Barcelona, Spain, to assess functional impairment focusing on the main problems experienced by the mentally ill, including bipolar patients. The initial version of the FAST included 56 items divided into 10 specific areas, such as autonomy, work functioning, cognitive functioning, finances, insight, social/marital life, acceptance/knowledge disorder, strategies to cope with symptoms, use of medication, and self-fulfilment. This version was studied in a pilot study with ten bipolar patients and ten healthy controls. After preliminary analysis, the scale was discussed in a meeting with experts from Spain, Brazil and England and several changes were made and some items were rejected. Then, the final version of the FAST was designed.

#### 3.2. Description

The FAST is an interviewer-administered instrument which is designed to be used by a trained clinician; the studied time frame refers to the last 15 days before assessment. It is a quite simple instrument, easy to apply and which only requires a short time to apply (see additional files 1 and 2). It comprises 24 items, which are divided among 6 specific areas of functioning:

1) Autonomy refers to the capacity of the patient of doing things alone and take his/her own decisions.

2) Occupational functioning refers to the capacity to maintain a paid job, efficiency of performing tasks at work, working in the field in which the patient was educated and earning according to the level of the employment position.

3) Cognitive functioning is related to the ability to concentrate, perform simple mental calculations, solve problems, learn new information and remember learned information.

4) Financial issues involve the capacity of managing the finances and spending in a balanced way.

5) Interpersonal relationships refer to relations with friends, family, involvement in social activities, sexual relations and the ability to defend ideas and opinions.

6) Leisure Time refers to the capacity of performing physical activities (sport, exercise) and the enjoyment of hobbies.

All of items are rated using a 4-point scale, 0 = no difficulty, 1 = mild difficulty, 2 = moderate difficulty and 3 = severe difficulty. The global score is obtained when the scores of each item are added up. The higher the score, the more serious the difficulties are, so FAST is actually measuring disability.

### 4. Psychometrics

We analysed the feasibility, internal consistency, concurrent validity, validity as a discriminative measure to detect difference between euthymic and acute patients, factorial analyses and test-retest reliability of the FAST. Except for test-retest reliability, the psychometric characteristics of the FAST are derived from the first administration of the questionnaire, including all the subjects who completed it in the analysis.

4.1. Feasibility is described as the percentage of patients who did not respond to the questionnaire in its entirety. It also includes the time spent in completing the instrument as a measure of how practical it may be for busy clinicians and for its inclusion in clinical trials and other studies.

4.2. Internal consistency reliability assesses the degree to which questions on an instrument measure the same underlying concept. The alpha internal consistency coefficient of reliability (Cronbach's Alpha) was used to examine the internal consistency of the FAST items in each domain and total scale. The correlation between each domain and the total scores was calculated.

4.3. Concurrent validity was studied considering GAF instrument and the score obtained on the FAST applying the Pearson correlation coefficient [21]. The GAF was chosen as the scale to assess concurrent validity of the FAST because it is probably the main instrument for assessing functional outcome in mental disorders.

4.4. The optimal point for the FAST was determined by means of ROC curves.

4.5. Test-retest reliability: Intra-class correlation coefficient was performed to assess test-retest reliability, 15 subjects were identified who had remained stable for at least one week, according to YMRS, HDRS-17 and GAF. These 15 subjects then participated in a Test-Retest reliability assessment one week later.

4.6. Validity as a discriminative measure to detect difference between euthymic and acute patients: the participants were stratified by severity of symptoms in euthymia, mania, and depression, as determined by a clinical assessment based on DSM-IV TR. An ANOVA analysis was used to evaluate whether the FAST total scores were sensitive to the severity of symptoms.

4.7. An orthogonal factorial analysis by matrix rotation was performed to describe the internal structure of each domain of FAST.

### 5. Statistical analysis

Statistical analysis was performed using SPSS for Windows – Version 12.0 (SPSS Inc., Chicago, IL, USa). Pearson's correlation coefficient was performed to examine the correlation between FAST and GAF scores. Internal consistency was analyzed using the Cronbach's alpha. Total scores of FAST of three groups (euthymic, manic or depressed) were compared using a one-way ANOVA. When ANOVA comparing more than two groups showed significant differences, the individual Tukey HSD test was performed. Intra-class correlation coefficient was performed to assess the reliability between test and retest. The rotation was performed using the Varimax method.

## Results

Seventy one euthymic, fourteen depressive and sixteen manic patients were enrolled to participate in the study. The mean age of the patients was 45 years (SD: 13.66, median 45.45, ranging from 22 to 82) and mean age of the controls was 49 years (SD:17.66, median 49.16, ranging from 22 to 81). 51.5% of patients and 57.4% of controls were women. There were no significant differences between the groups in demographic baseline assessment. Table 1 describes the main socio-demographic and clinical characteristics of the sample. in.

**Table 1:** Demographic and clinical variables

	Bipolar patients		Control group	
	Mean	SD	Mean	SD

Age (*p* value = 0.31)	45.45	13.66	49.16	17.66
Age of onset	27.82	11.84		
Chronicity	17.92	11.51		
Total episodes	12.35	15.06		
Manic episodes	3.43	4.61		
Hypomanic episodes	3.00	9.43		
Depressed episodes	5.60	6.67		
Hospitalization	1.93	1.96		
Suicide attempts	1.44	0.49		
HDRS	5.41	7.17		
YMRS	4.08	6.67		
GAF	63.90	19.05		

	n	%	n	%

Sex (*p* value = 0.14)				
Female	52	51.5	35	57.4
Male	49	48.5	26	42.6
Bipolar type I	89	88.3		
Bipolar type II	11	10.7		
Bipolar NOS	1	1		

Among the 101 bipolar patients, mood stabilizing agents were the most commonly prescribed agents (81.6%), including lithium (59.2%), valproate (13.6%) and carbamazepine (9.7%); 12.6 % received lamotrigine; 63.1% antipsychotics, 30.1% antidepressants and 48.5% anxiolytic-sedatives.

All items of the FAST were answered by 99% the patients (n = 100) in every test session. The mean time spent in completing the instrument was 6.00 minutes (ranging from 2 to 12; SD: 2.79) for the total sample, 5.69 (SD: 2.67) for euthymic, 5.29 (SD: 1.64) for depressive and 7.25 (SD: 3.26) for manic patients. No difference was found between groups (F = 2.65; p = 0.075).

The internal consistency coefficient obtained was high, Cronbach's alpha of 0.909, for the total scale, indicating that the items are sufficiently homogeneous. The FAST also showed high internal consistency on each of the twenty-four items.

**Figure 1:**
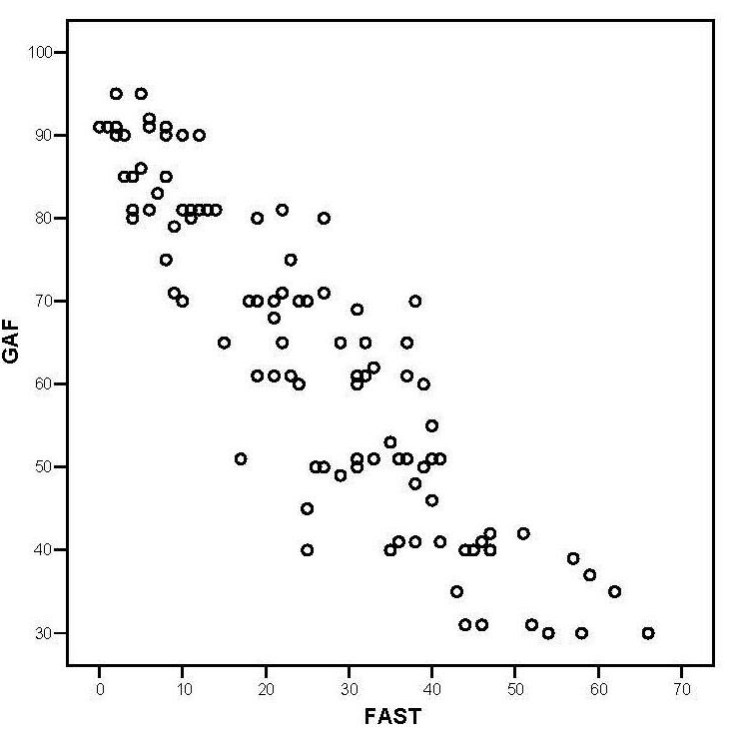
A Pearson correlation between scores of GAF and scores of FAST. * Negative correlation between total scores of FAST and GAF (r = -0.903; p < 0.001).

Concurrent validity based on functional impairment according to GAF scale showed a highly significant negative correlation (r = -0.903; p < 0.001). This result indicates that patients with good functioning assessed using the FAST obtained higher scores on the GAF scale, as shown in Figure 1. Accordingly GAF scores indicated adjustment whereas FAST scores showed disability.

We analysed the scale's discriminant capacity between patient and controls by means of the diagnostic performance or ROC curve. The area under the curve was 0.86, 95%CI: (0.809, 0.917) which, being close to 1, indicates a good capacity. The discriminant capacity study indicates that a score above 11 obtains the best balance between sensitivity (72%) and specificity (87%). The mean total FAST score in patients and the control group were 25.43 (0–66; SD:16.31) and the 6.07(0–20; SD: 4.72) respectively.

Intra-class correlation coefficient was 0.98 (p < 0.01), as shown in table 2. The YMRS, HDRS-17 and GAF were assessed during test and retest to verify stability on the mood states of patients. These results indicate that the stability of the FAST would not be altered by natural mood variations in the patients' condition.

**Table 2:** Test-Rest reliability of the FAST

					Intraclass	F	gl	p	t test	F	gl	p
N = 15	mean	SD	Mean	SD	correlation							
FAST	37.73	16.79	34.87	16.14	0.98	40.98	14	0.0001	0.48	0.006	28	0.94
GAF	49.40	17.80	52.53	16.74	0.95	18.63	14	0.0001	-0.50	0.29	28	0.60
HDRS	10.53	10.21	9.87	9.99	0.87	7.59	14	0.0001	0.18	0.047	28	0.83
YMRS	9.80	10.10	9.00	8.44	0.93	14.23	14	0.0001	0.24	1.47	28	0.24

**Figure 2:**
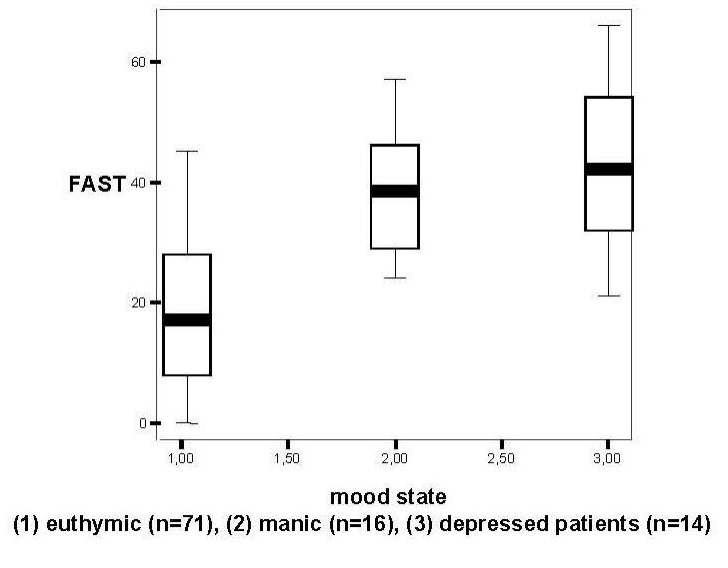
FAST scores across different mood states in bipolar patients. * Significant difference between euthymic patients (18.55; F = 35.43; p < 0.001) and acute patients (manic mean: 40.44±9.15 and depressed mean: 43.21±13.34).

Validity was assessed using a discriminant measure to detect difference between euthymic and acute patients. The mean scores of FAST were lower in euthymic (18.55 ± 13.19; F = 35.43; p < 0.001) patients, as compared with manic (40.44 ± 9.15) and depressed patients (43.21 ± 13.34) as shown in figure 2.

The study of the internal structure of the FAST, after rotation (using Varimax method), determined a five-factor structure, as shown in table 3. In this analysis it was observed that the interpersonal relationship and leisure time domains are loading on the same factor.

**Table 3:** Factorial loading on the FAST

FAST*	1	2	3	4	5
EF 5	0.938				
EF 9	0.908				
EF 7	0.902				
EF 6	0.882				
EF 8	0.852				

EF 17		0.741			
EF 18		0.628		0.437	
EF 21		0.596			
EF 24		0.577	0.544		
EF 23		0.552			
EF 20		0.466			
EF 19		0.403			

EF 12					
EF 13			0.746		
EF 14			0.694		
EF 11			0.668		
EF 10			0.480		

EF 4				0.741	
EF 1				0.724	
EF 3			0.407	0.693	
EF 2				0.531	

EF 22					
EF 16					0.897
EF 15					0.870

* Coefficient less than 0.42 are omitted

## Discussion

High rates of functional impairment among bipolar patients, even amongst patients whose symptoms have remitted, have been documented in numerous studies [4,7,22-25]. Cognitive impairment, work impairment, household duty difficulties, interpersonal relationship difficulties, leisure time difficulties, and sexual problems are the main functioning problems presented by patients [6,7,13,14,17]. However, the majority of instruments available to date are very lengthy and have been focused on global or limited measures of functional recovery, rather than examining specific, discrete areas of psychosocial activity [4,7]. Therefore, the development of specific instruments to assess the functional outcome in BD is still an unmet need [13,14]. Within this context, the FAST could become particularly useful.

The FAST comprises twenty-four items that assess six specific areas of functioning such as autonomy, occupational functioning, cognitive functioning, financial issues, interpersonal relationships and leisure time. The FAST presents advantages due to the simplicity of the instrument, the ease of its application and the time frame required for its implementation. In addition, the FAST shows high feasibility, a quality that makes it applicable in both clinical practice and in research settings. The instrument is also available in three languages, a Spanish version, a Portuguese and an English version.

The psychometric properties of the FAST showed high internal consistency, where the total items had a Cronbach's alpha above 0.9. In addition, we found a strong concurrent validity, and discriminant validity. The test-retest reliability, which only featured patients with stable mood states, showed very similar results. The FAST showed to be a sensitive instrument for the detection of different mood states, and this was supported by the fact that euthymic patients showed functioning results twice as higher than depressed and manic patients. Previous studies showed moderate to marked impairment in specific areas of functioning and the persistence of depressive symptoms was also significantly associated with impairment [3,5,12,17,24]. Strakowski et al. (2000)[7] reported that functional recovery, and in particular, interpersonal relationships recovery was associated with recovery from manic symptoms. The present study showed that the severity of symptoms was associated with higher scores of FAST and poorer functioning.

In this study, we suggest a putative cut-off point higher than 11, because this value improved the test's discriminant properties, obtaining a sensitivity of 72% and a specificity of 87%. Using this cut-off point, the total FAST score was 25.43 for patients (0–66; SD: 16.31) and 6.07 for healthy controls (0–20; SD: 4.72); only five healthy controls showed a score superior to11. These results are consistent with previous studies, suggesting that functional impairment is not restricted to acute episodes and remitted patients may show functional impairment despite symptomatic recovery [5-7,24,26].

As regard to concurrent validity, the FAST showed a strong negative correlation with the GAF scale, which is the main instrument for assessing the current level of functioning [13,14]. The GAF gives ratings from 0 to 100, which specifies anchors for quantification of overall psychosocial functioning adjustment, where the higher scores of GAF represents better psychosocial functioning [12,13,27]. In opposition to the GAF, the FAST assesses specific domains of functioning and also identifies the level of impairment in each area; higher scores represent higher disability thus a negative correlation was actually expected.

Available instruments to assess functioning include the WHO-DAS-II, SF-36, SAS and LFQ scale. WHO-DAS is an instrument developed by The World Health Organization, which can predict disability related outcomes. However, the instrument is an extensive interview, which limits its use in clinical practice and it also assesses numerous physical domains which are less relevant than mental domains to psychiatric patients in general and bipolar patients in particular [28]. The SF-36 is a self-reported instrument, consisting of 36 items and 8 subscales measuring the domains of physical and social functioning, as well as general and emotional health [23,29]. The Social Adjustment Scale (SAS) is a comprehensive instrument to assess multiple domains of social functioning and its length is a barrier to use in screening or routine assessment. In addition, it is most commonly used in the area of depression treatment [30]. The Life Functioning Questionnaire (LFQ) is a brief questionnaire which assesses duties at home, leisure activities (family/friends) and duties at work [28]. However, all the scales above did not assess important areas of functioning such as cognitive functioning and finances. Furthermore, the use of self-reported scales to assess functional impairment in psychiatric illness, particularly bipolar patients, is not reliable because of the extensive psychopathology of these patients may make them more prone to over-estimate or under-estimate their own disability [5,31,32]. At this moment, the instruments available were not developed to measure the health problems particularly associated with BD. This results in a lack of standardisation of these instruments which gives difficulties in understanding the results. In addition, the validation of the scales in other languages, in particular, Spanish version is very important because Spanish is spoken by over 352 million people worldwide [14,18,33-35]. In this context, the FAST was designed considering the main difficulties experienced by psychiatric patients reported in the literature and previous scales including those expressed by bipolar patients and it is promoting a new option to assess functional impairment in specific domains that may be affected in bipolar patients.

## Conclusion

In conclusion, the FAST showed strong psychometric properties and it was sensitive to different mood states. In addition, it is a simple interviewer-administered instrument, which is easy to apply, only requires a short period of time for implementation, and it is now available to be used in the clinical practice and investigation settings. The FAST promotes the assessment of specific domains of functioning impairment in bipolar disorder patients. Therefore the use of FAST may be instrumental in the assessment of supplemental interventions targeting rehabilitation/functional enhancement of BD patients. FAST may also be useful in assessing the effect of pharmacologic and psychosocial interventions on functioning of psychiatric patients, being valid, reliable and user-friendly in the field of bipolar disorder.

## Competing interests

The author(s) declare that they have no competing interests.

## Supplementary Material

Additional file 1 **Prueba Breve de Evaluación del Funcionamiento (spanish version of the scale)**. Click here for file

Additional file 2 **Functioning Assessment Short Test (english version of the scale)**. Click here for file
