# Supplementary material for: Validity and reliability of the Functioning Assessment Short Test (FAST) in bipolar disorder
Source: Clin Pract Epidemiol Ment Health. 2007 Jun 7;3:5. doi: 10.1186/1745-0179-3-5 (PMC1904447; doi:10.1186/1745-0179-3-5)
Supplement: Additional file 1 — Prueba Breve de Evaluación del Funcionamiento (spanish version of the scale). [file 1745-0179-3-5-S1.doc]

**PRUEBA BREVE DE EVALUACIÓN DEL FUNCIONAMIENTO (FAST)**

**¿Cuál es el grado de dificultad del paciente en relación con los siguientes aspectos?**

| **AUTONOMIA**  1. Encargarse de las tareas de la casa  2. Vivir solo  3. Hacer la compra  4. Cuidar de sí mismo (aspecto físico, higiene...) | (0) (1) (2) (3)  (0) (1) (2) (3)  (0) (1) (2) (3)  (0) (1) (2) (3) |
| --- | --- |
| FUNCIONAMIENTO LABORAL 5. Realizar un trabajo remunerado  6. Acabar las tareas tan rápido como era necesario  7. Trabajar en lo que estudió  8. Cobrar de acuerdo con el puesto que ocupa  9. Alcanzar el rendimiento previsto por la empresa | (0) (1) (2) (3)  (0) (1) (2) (3)  (0) (1) (2) (3)  (0) (1) (2) (3)  (0) (1) (2) (3) |
| FUNCIONAMIENTO COGNITIVO10. Concentrarse en la lectura, película 11. Hacer cálculos mentales  12. Resolver adecuadamente un problema  13. Recordar el nombre de gente nueva  14. Aprender una nueva información | (0) (1) (2) (3)  (0) (1) (2) (3)  (0) (1) (2) (3)  (0) (1) (2) (3)  (0) (1) (2) (3) |
| FINANZAS15. Manejar el propio dinero 16. Hacer compras equilibradas | (0) (1) (2) (3)  (0) (1) (2) (3) |
| RELACIONES INTERPERSONALES17. Mantener una amistad 18. Participar en actividades sociales  19. Llevarse bien con personas cercanas  20. Convivencia familiar  21. Relaciones sexuales satisfactorias  22. Capaz de defender los propios intereses | (0) (1) (2) (3)  (0) (1) (2) (3)  (0) (1) (2) (3)  (0) (1) (2) (3)  (0) (1) (2) (3)  (0) (1) (2) (3) |
| **OCIO** 23. Practicar deporte o ejercício 24. Tener una afición | (0) (1) (2) (3)  (0) (1) (2) (3) |

Interrogue al paciente respecto a las áreas de funcionamiento que se especifican a continuación, utilizando la siguiente escala: (0): Ninguna, (1): Poca, (2): Bastante o (3): Mucha.
