# Supplementary material for: Validity and reliability of the Functioning Assessment Short Test (FAST) in bipolar disorder
Source: Clin Pract Epidemiol Ment Health. 2007 Jun 7;3:5. doi: 10.1186/1745-0179-3-5 (PMC1904447; doi:10.1186/1745-0179-3-5)
Supplement: Additional file 2 — Functioning Assessment Short Test (english version of the scale). [file 1745-0179-3-5-S2.doc]

**FUNCTIONING ASSESSMENT SHORT TEST (FAST)**

**To what extent is the patient experiencing difficulties in the following aspects?** Ask the patient about the areas of difficulty in functioning and score according to the following scale: (0): no difficulty, (1): mild difficulty, (2): moderate difficulty, (3): severe difficulty

| **AUTONOMY**  1. Taking responsibility for a household  2. Living on your own  3. Doing the shopping  4. Taking care of yourself (physical aspects, hygiene) | (0) (1) (2) (3)  (0) (1) (2) (3)  (0) (1) (2) (3)  (0) (1) (2) (3) |
| --- | --- |
| **OCCUPATIONAL FUNCTIONING**  5. Holding down a paid job  6. Accomplishing tasks as quickly as necessary  7. Working in the field in which you were educated  8. Occupational earnings  9. Managing the expected work load | (0) (1) (2) (3)  (0) (1) (2) (3)  (0) (1) (2) (3)  (0) (1) (2) (3)  (0) (1) (2) (3) |
| **COGNITIVE FUNCTIONING**  10. Ability to concentrate on a book, film  11. Ability to make mental calculations  12. Ability to solve a problem adequately  13. Ability to remember newly-learned names  14. Ability to learn new information | (0) (1) (2) (3)  (0) (1) (2) (3)  (0) (1) (2) (3)  (0) (1) (2) (3)  (0) (1) (2) (3) |
| **FINANCIAL ISSUES**  15. Managing your own money  16. Spending money in a balanced way | (0) (1) (2) (3)  (0) (1) (2) (3) |
| **INTERPERSONAL RELATIONSHIPS**  17. Maintaining a friendship or friendships  18. Participating in social activities  19. Having good relationships with people close you  20. Living together with your family  21. Having satisfactory sexual relationships  22. Being able to defend your interests | (0) (1) (2) (3)  (0) (1) (2) (3)  (0) (1) (2) (3)  (0) (1) (2) (3)  (0) (1) (2) (3)  (0) (1) (2) (3) |
| **LEISURE TIME**  23. Doing exercise or participating in sport  24. Having hobbies or personal interests | (0) (1) (2) (3)  (0) (1) (2) (3) |
